# Simultaneous Bilateral Spontaneous Pneumothorax Revealed Birt-Hogg-Dubè Syndrome

**DOI:** 10.1155/2015/916039

**Published:** 2015-09-01

**Authors:** Alessandro Tamburrini, Francesco Sellitri, Federico Tacconi, Francesco Brancati, Tommaso Claudio Mineo

**Affiliations:** ^1^Thoracic Surgery Division, Tor Vergata University, Viale Oxford 81, 00133 Rome, Italy; ^2^Department of Medical, Oral and Biotechnological Sciences, Gabriele D'Annunzio University, Chieti, Italy; ^3^Medical Genetics Unit, Policlinico Tor Vergata University Hospital, Rome, Italy

## Abstract

Simultaneous bilateral spontaneous pneumothorax is a very rare clinical event, comprising approximately 1% of all spontaneous pneumothoraces. Clinical signs and symptoms may vary from mild chest pain and dyspnea to severe respiratory failure; nevertheless immediate treatment is mandatory as this condition can deteriorate and progress to tension pneumothorax. An underlying lung disease has been commonly described; in most istances primary or secondary tumors, interstitial diseases, and infectious diseases. Birt-Hogg-Dubè syndrome is a rare inherited disorder clinically characterized by multiple fibrofolliculomas, renal tumors, lung cysts, and, in ~24% of the patients, occurrence of spontaneous pneumothorax. In this case, we firstly report the concurrence of these rare conditions, as a patient presenting a simultaneous bilateral spontaneous pneumothorax was diagnosed with Birt-Hogg-Dubè syndrome based on the typical radiological findings and genetic testing of the folliculin gene located on chromosome 17.

## 1. Introduction

Simultaneous bilateral spontaneous pneumothorax (SBSP) is a very rare condition, accounting for ~1% of all spontaneous pneumothoraces [1]. Most of the reported cases are associated with underlying lung diseases. Likewise, Birt-Hogg-Dubè (BHD) syndrome is a rare autosomal dominant disorder characterized by multiple fibrofolliculomas, lung cysts, pneumothorax, and renal tumors [2]. We herein report a unique case of a young adult in whom the occurrence of simultaneous bilateral spontaneous pneumothorax led to the diagnosis of BHD syndrome.

## 2. Case Report

**Figure 1:**
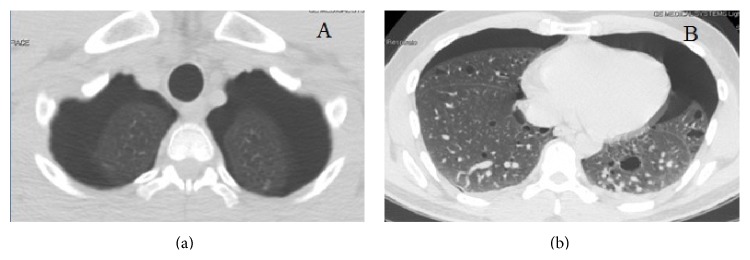
Chest CT-scan showing the apical (a) and the basal (b) aspect of the simultaneous bilateral spontaneous pneumothorax, as well as the multiple lung cysts (b).

**Figure 2:**
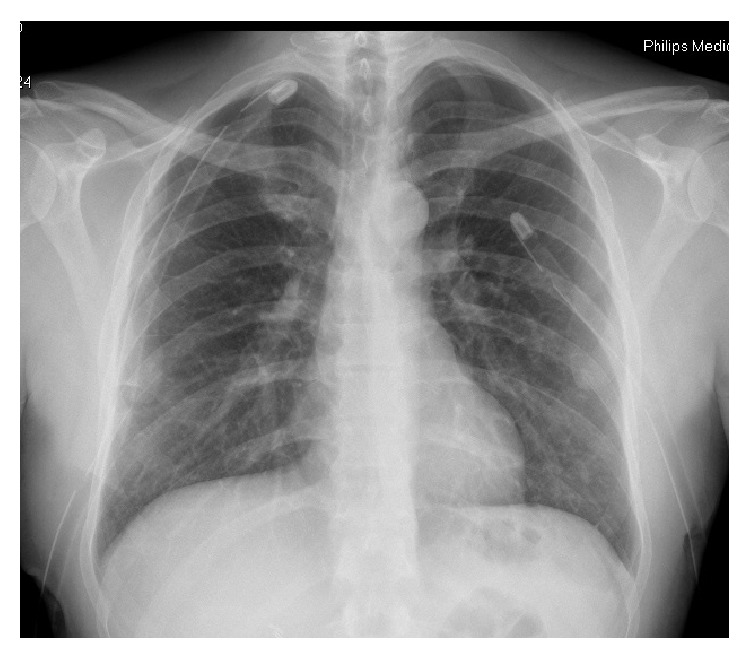
Chest X-ray showing reexpansion of the lungs following bilateral chest tube insertion.

**Figure 3:**
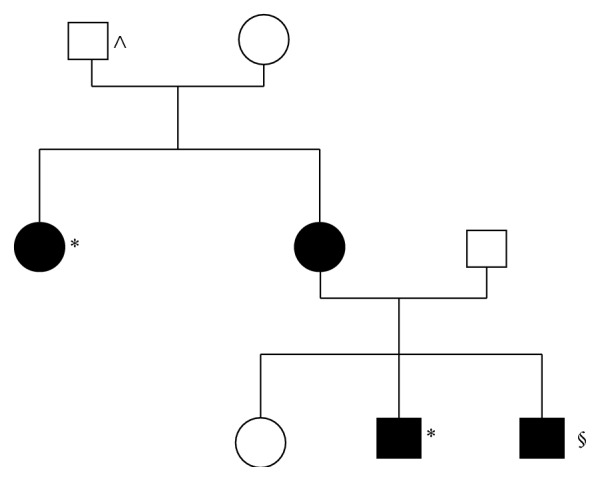
The patient's family tree. Black = affected by BHD syndrome, presence of c.1285dup mutation in the FLCN; ∧ = possible BHD syndrome, genetic testing not available; *∗* = being symptomatic for pneumothoraces; § = being symptomatic for renal cancer.

A previously healthy 43-year-old man was admitted to the Emergency Room complaining of left-sided chest pain and increasing breathlessness. The patient was afebrile, mildly tachycardic, and normotensive. Breath sounds were decreased bilaterally and SpO_2_ was 90%. Oxygen administration was immediately started and a chest CT-scan was obtained, revealing a simultaneous bilateral pneumothorax which was larger on the left side. In addition, numerous elliptically shaped lung cysts of various sizes were present bilaterally, predominantly distributed in the basilar medial and lateral regions, and in the subpleural regions (Figure 1). The patient was treated with bilateral chest tube insertion, and the X-ray showed a successful reexpansion of both lungs (Figure 2). No persistent air leaks were demonstrated after 48 hours. The patient was scheduled for a single-stage bilateral video-assisted thoracoscopy where a small wedge resection was performed on the left lung while mechanical pleurodesis was performed on both sides. The postoperative period was uneventful and the patient was discharged after 72 hours. Radiological imaging was highly suggestive of a multiple cystic lung disease. Moreover, family history revealed that a patient's aunt was diagnosed several years earlier with bullous emphysema and had 2 episodes of pneumothorax, while his deceased grandfather suffered from an unspecified pulmonary disease. Given all these distinguishing findings, we suspected a BHD syndrome. The diagnosis was confirmed after genetic testing, which revealed the c.1285dup (p.H429PfsX27) truncating mutation on exon 11 in the folliculin (FLCN) gene located on chromosome 17 [2]. At 18-month follow-up, no recurrence of pneumothorax has been observed. Following the surgical procedure, genetic testing was performed on all patient's family members. The same mutation was found in the patient's mother and in her sister, as well as in the patient's brother who was also later diagnosed with renal cancer (Figure 3).

## 3. Discussion

Simultaneous bilateral spontaneous pneumothorax is of rare occurrence. Clinical presentation can be variable, with signs and symptoms fluctuating from mild dyspnea and chest pain to severe shortness of breath and respiratory failure. Although a lung collapse of less than 60% has been generally reported with SBPS, early diagnosis and treatment by bilateral chest drainage are mandatory, in order to avoid possible life-threatening progression to tension pneumothorax [1].

The only large review available reports 77 patients with SBSP. In this series, Sayar et al. [1] observed an underlying lung disease causing the SBSP in ~65% of the cases. Proliferation of mesenchymal cells was the leading cause of SBSP. Within the list, sarcomas of various origins were mostly reported (12 cases), followed by histiocytosis X (4 cases), lymphoma (3 cases), and a variety of interstitial lung diseases. Despite their significantly higher overall prevalence, both primary lung carcinoma and pulmonary metastases of epithelial malignancies caused SBSP in only 5 patients. Infectious diseases were reported in over 15% of the patients, with either cavitary or military tuberculosis covering nearly half of the cases. Congenital diseases were reported as well, with 3 cases of cystic fibrosis, 2 cases of Marfan's syndrome, and only single case for endometriosis, Alport's syndrome, and lung cyst. Anorexia nervosa and chronic obstructive pulmonary disease have been described as well [1]. Recently, cases of SBPS due to congenital [3] or iatrogenic [4] pleuropleural communication have also been reported, while over 35% of patients with SBSP do not present any specific lung disease [1]. In a recent analysis by Lee et al. [5], SBPS was also significantly associated with lower BMI and with the abnormal presence of subpleural blebs/bullae.

Birt-Hogg-Dubè syndrome is a very rare disorder and unfamiliar to many physicians, so the diagnosis can be frequently delayed. It is well known that the occurrence of spontaneous pneumothorax is a possible clinical manifestation of BHD syndrome, with a high risk of recurrence [2]. Nevertheless, SBSP has never been described among the typical clinical features of this disease [2]. Toro et al. [6] evaluated 198 patients with BHD syndrome and reported a 24% incidence of spontaneous pneumothorax, but no episodes of SBSP were described. Verhaert [7] instead did report a case of bilateral pneumothorax in a patient with BHD syndrome, but the pneumothoraces had occurred within a distance of few days. In our case, pneumothoraces occurred spontaneously and simultaneously in both lungs. Differential diagnosis among the most common cystic lung diseases like Lymphangioleiomyomatosis (LAM), pulmonary Langerhans cell histiocytosis (LCH), and lymphoid interstitial pneumonia (LIP) had to be considered as well [8]. Certain characteristics of the pulmonary cysts in our patient were however highly suspicious for BHD syndrome. They were in fact multiple and bilateral, mainly elliptical and lentiform shaped, well defined air-filled cysts, homogeneously distributed among the basilar, medial, and subpleural regions, differing from the typical apical location seen in cases of emphysematous blebs and bullae [2]. Patients with LAM are almost exclusively women [8], and their lung cysts are more diffusely distributed in both upper and lower lobes; they present a rounder shape and often a bigger size [2]. In LCH, a combination of both nodules and cysts is usually present, with the latter being often irregularly shaped and predominantly distributed in the upper- and mid-lung zones [2]. Lastly, LIP is usually associated with other autoimmune diseases, lung cysts are associated with ground-glass opacities, nodules, and septal thickening, and a distinctive inheritance of the disorder cannot be found [2]. Nevertheless, confirmation of BHD syndrome diagnosis by genetic testing is mandatory. In our case, one of the most common [9] truncating mutations of the FLCN gene located on the chromosome 17 (c.1285dup on exon 11) was found in the patient and in three of his family members.

Initial pneumothorax in BHD patients is usually managed with conservative measures (observation, aspiration, and tube thoracostomy), while VATS procedures are typically reserved for recurrent or nonhealing cases [2]. In our circumstances instead, an early thoracoscopic approach following bilateral chest tube insertion was the therapeutic option of choice, as advocated by Sayar et al. [1]. They propose pleurectomy and/or pleurodesis as a crucial treatment component in the management of SBPS cases, and no patients in their series had recurrent pneumothorax during hospital stay or were readmitted due to any complication [1]. Similarly, no recurrence was observed in our case after 18 months from discharge.

## 4. Conclusion

SBPS and BHD syndrome are both extremely rare and their association has never been previously reported. One should be aware that SBPS can occur and must be immediately treated. If multiple bilateral lung cysts are also present, BHD syndrome is a reasonable diagnosis to suspect. A family history of recurrent pneumothorax and/or renal cancer as well as a typical distribution and radiological aspects of the pulmonary cysts can be helpful in distinguishing BHD syndrome from other cystic lung diseases.
